# Case Report: The Parkes-Weber syndrome in the patient who underwent coronary surgery

**DOI:** 10.3389/fcvm.2025.1479811

**Published:** 2025-07-15

**Authors:** Nemanja Milosevic, Bogdan Okiljevic, Slobodan Micovic, Milovan Bojic, Igor Zivkovic

**Affiliations:** ^1^ Cardiac Surgery Clinic, Institute for Cardiovascular Diseases Dedinje, Belgrade, Serbia; ^2^ Faculty of Medicine, University of Belgrade, Belgrade, Serbia

**Keywords:** Parkes-Weber syndrome, CABG, vascular anomaly, congenital anomalies, arteriovascular fistula

## Abstract

Parkes-Weber Syndrome is a rare congenital vascular abnormality characterized by aneurismatic illness of blood arteries in the afflicted limb, as well as hypertrophy, ulceration, ischemia, and high-output heart failure. Imaging investigations are required to provide a diagnosis, with contrast arteriography being the gold standard. The majority of treatment options are endovascular, with surgical excision for arteriovenous malformations and limb amputation as alternatives. We describe a 73-year-old male patient with mainly asymptomatic PWS, coronary disease, and borderline EF (45%) who had CABG surgery. In individuals with established CAD and other cardiac disorders, it is critical to identify additional diseases or syndromes that might have a compounding effect on the heart, such as PWS and high-output heart failure.

## Introduction

Parkes-Weber syndrome (PWS) is a group of complicated congenital vascular anomalies that include capillary, venous, lymphatic, and arteriovenous malformation (AVM) in the overgrown limb. PWS may affect both the upper and lower extremities, including the pelvic vessels (1, 2). The RASA1 mutation was identified as the aberrant gene involved in PWS development (3). This health condition typically manifests as overgrowth of the affected limb, high-output heart failure, chronic venous ulcers, and distal arterial ischemia. In addition to conventional symptoms, some individuals present with spinal AVM and aneurysms in the largest arteries of the afflicted limb. Grayscale and Doppler ultrasonography are the primary diagnostic tools; subsequent imaging tests include magnetic resonance imaging (MRI) and contrast-enhanced computer tomography (CT). Contrast arteriography is the gold standard for diagnosis, however it may be reserved for patients who require embolectomy. Treatment methods for this type of disease include percutaneous coil embolization, surgical AVM excision, amputation, and, in rare cases, stent-graft implantation (1).

## Case presentation

**Figure 1:**
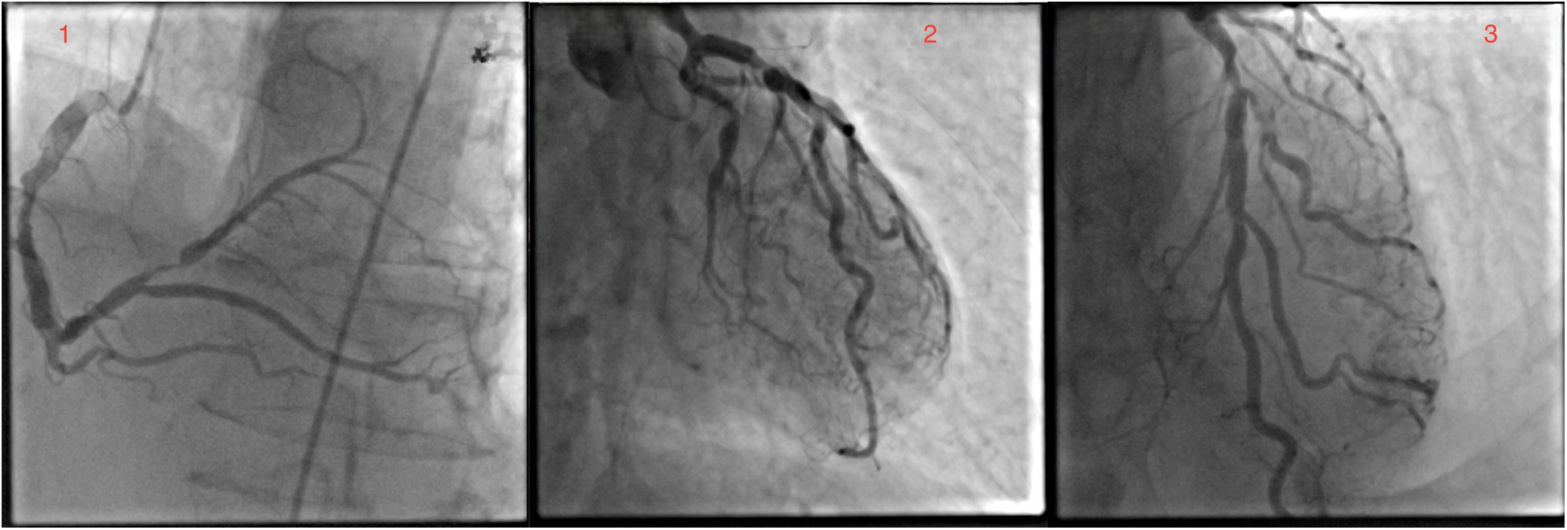
Coronary angiography revealed significant left main and three-vessel disease.

A 73-year-old white male patient had been admitted to the hospital for elective coronary artery bypass grafting. On admission, the patient reported anginous pain at rest without propagation and passes spontaneously, as well as exhaustion from moderate physical effort. In addition, the patient reported losing consciousness on multiple occasions without other neurological signs and symptoms. Risk factors: hypertension, dyslipidemia, former smoker (smoked around 40ty years for 10–20 cigarettes a day), ceased smoking after being hospitalized for MI, and family history of cardiovascular disease, mother and brother dying from myocardial infarction at age 72 and 60 years old, respectively. The patient's everyday medications were: acetylsalicylic acid, clopidogrel, bisoprolol, isosorbide mononitrate, furosemide, KCl, trimetazidine, and rosuvastatin. ECG on admission was: sinus rhythm with 72 bpm, with reduced R wave in inferior leads and negative T wave. The most notable physical manifestation was severely dilated veins in the right arm and a port wine stain on the protrusion of the deltopectoral groove. Further physical examination revealed collapsible veins and pulsing swelling with a trill in the cubital fossa in a projection of the ulnar artery and brachial vein, as well as many pulsating swellings (in the center) on the inner side of the upper arm (Supplementary Video S1). The patient reports having a noticeable vein patern of the right arm from a young age (12 years old) and slowly progressing through life without any physical impairment to quality of life, as such, he never sought any medical help. Four months prior to admission at our Institute, the patient was admitted to cardiology at a local general hospital under suspicion of acute coronary syndrome. At that time, ECG showed sinus rhythm, with inverted T wave in D2, D3, and aVF leads and the highest value of hsTroponin 165 ng/L. In the same hospitalization, coronary angiography and fFR were done and found: ectatic left main with distal stenosis of 60%, ectatic LAD with medial stenosis of 70%, and proximal suboclusion of the diagonal artery, as well as ectatic LCx, with ostial stenosis of 75% and marginal artery with stenosis of 70%; dominant, ectatic and with diffusely atherosclerotic RCA, and PL with proximal stenosis of 90% (Figure 1). Fractional flow reserve (FFR) of LAD was 0.79. The transthoracic echocardiography has shown a normal mitral valve with partially fibrous cusps, normal flow (0.6 m/s), and mitral regurgitation of 1+, a normal tricuspid valve, aortic valve with preserved separation and coaptation with the normal flow (1 m/s), an akinetic basal part of the inferior wall, an akinetic basal part of the posterior wall, and an akinetic base of the ventricular septum, end-diastolic diameter 48 mm, end-systolic diameter 35 mm, and ejection fraction 45%.

**Figure 2:**
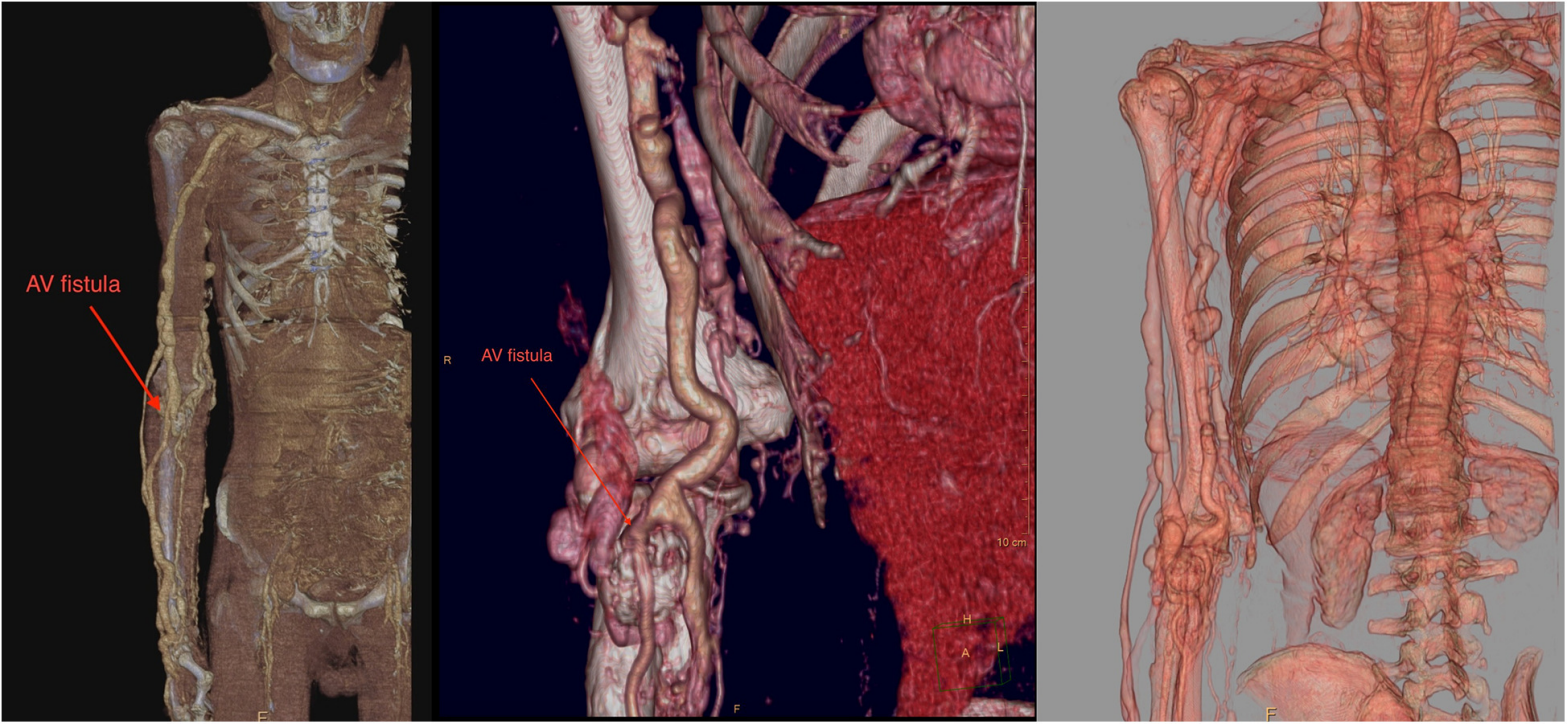
A contrast computer tomography of the upper arm revealed dilated axillary and brachial arteries with arteriovenous malformation (red arrow).

**Figure 3:**
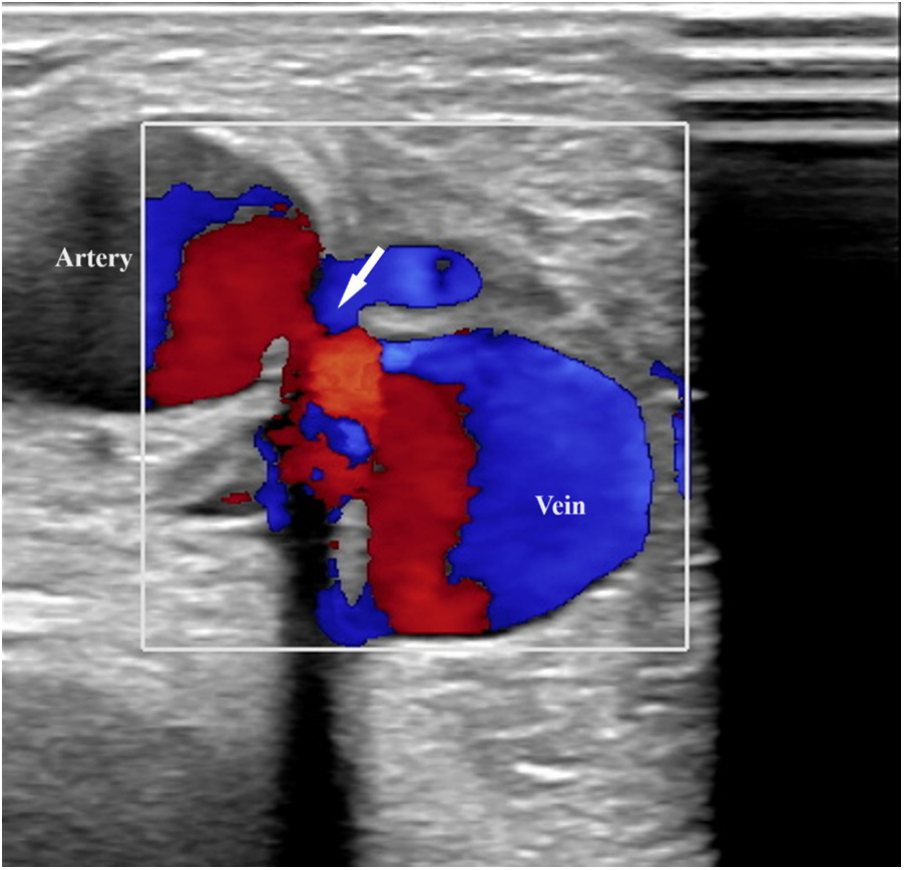
Doppler sonography of the right arm revealed an arteriovenous fistula (in the middle) on the inner side of the upper arm.

Upon admission to our institute, a CT scan of supra-aortic arteries and ascending and descending aorta was done. A CT scan of supra-aortic arteries revealed a diffusely dilated right axillar artery with a diameter of 19 mm, as well as a 21 × 15 mm aneurysm 46 mm from the vertebral artery's exit. CT scans of the ascending and descending aorta revealed a segmentally calcified ascending aorta with a diameter of 37 mm and arcus aortae with a diameter of 28 mm, as well as an aneurysm of abdominal aorta in the infrarenal segment, measuring 39 mm in diameter and 95 mm in length. CT scans reveal the existence of an arteriovenous fistula in the brachial artery (Figure 2). Doppler sonography of the right arm was performed, which was collaborated with CT findings of the same arm, the presence of an arteriovenous fistula (in the middle) on the inner side of the upper arm, and the presence of an arteriovenous fistula in the cubital fossa in an ulnar artery projection (Figure 3). Due to severe coronary disease, unprotected left main, and three-vessel disease, the heart team recommended surgical revascularization of the left and right system of coronary arteries. Standard on-pump CABG surgery in general anesthesia was done, with central cannulation of the aorta and through the right atrium. The anterograde infusion of cold blood del Nido cardioplegia stopped the heart. The surgical team decided on the use of a free mammary artery graft for LAD revascularization due to AV communication, and suspicion of steal syndrome. The right coronary artery and circumflex coronary artery were revascularized using a saphenous vein graft.

After an uneventful postoperative recovery, the patient was discharged on the 5th postoperative day without new changes in ECG and echocardiography, with the left ventricular ejection fraction remaining at 45%. Daily medication being acetylsalicylic acid, clopidogrel, bisoprolol, ramipril, torasemide, KCl, dapagliflozin, and rosuvastatin.

At 30 days follow-up, the patient was cardiopulmonary stable, without anginal pain, and tolerated effort well. ECG sinus rhythm with 66 bpm and without any changes since discharge. The patient reports an improvement in general condition without any significant events. At the 7-month follow-up, the patient reports no improvement in exercise tolerance, without angina or other heart symptoms. A heart echocardiogram found a slightly improved EF of 50%, an increase of LV, with an end-diastolic diameter of 57 mm and an end-systolic diameter of 37 mm, without any other differences from preoperative echo.

## Discussion

Parkes-Weber syndrome is a rare congenital disease characterized by severe vascular malformations, such as arteriovenous fistulas, which can induce limb swelling and high-output heart failure, as well as diminished quality of life and shortened life span (1). The RASA1 mutation was identified as the aberrant gene involved in PWS development (2).

Despite the low incidence of Parkes-Weber syndrome, it is essential to evaluate clinical indicators and the possibility of arteriovenous malformations. After combining Doppler ultrasonography and other imaging examinations to establish the diagnosis of PWS, a multidisciplinary evaluation is necessary, especially in the event of pre-existing cardiac illness, such as coronary artery disease, in light of potential heart failure. Due to significant AV malformation and potential problems with circulation through the internal mammary artery, in this case, the surgical team decided to use the mammary artery like a free graft to prevent potential steal syndrome after surgical revascularization.

According to Banzic et al., 15 (31.3%) patients suffered high-output cardiac failure as a result of significant AV shunt. There was no known gender preponderance (female-male ratio: 1:1), however, 10 (66.7%) patients were 16 years old or younger. Furthermore, high-output heart failure was documented in half of patients with upper limb involvement (3/6) and in more than one-fourth of cases with affected lower limbs (12/42), described patients with heart failure and PWS did not have significant coronary disease (3). Ahead of the surgery, our patient showed a modestly decreased EF of 45%, an akinetic basal part of the inferior wall, an akinetic basal part of the posterior wall, and a base of the ventricular septum, all of which were consistent with a past medical history of myocardial infarction. There were no symptoms of ischemic heart disease after interventions. Cardiac MRI should be considered as an option in determining myocardial viability before revascularization, as well as determining the etiology of heart failure (4, 5). According to ESC guidelines, CMR is recommended in determining the etiology of heart failure. CMR with Late gadolinium enhancement (LGE) should considered in DCM to distinguish between ischemic and non-ischemic myocardial damage (5).

Selvanayagam et al. reported that delayed-enhancement (DE/LGE) CMR is a potent predictor for myocardial viability after surgical revascularization (6). As such, it ought to be considered as a part of the perioperative assessment of all patients considered for myocardial revascularization, especially in the case of high-output HF caused by arterio-venous shunting (and any other etiology) combined with CAD and myocardial infarction, where CMR should also be part of the follow-up to determine how successful is the management of CAD and HF. Particularly in the case of any treatment of AV fistulas (embolization, surgical removal, stent implantation…) because of the possible existence of more AV fistulas distal to the original, which can mature over time and lead to the reappearance of PWS and HF symptoms (1).

The treatment aims are to enhance patients’ quality of life and reduce the risk of complications such as distal arterial ischemia, venous ulcerations, high-output heart failure, and excessive limb enlargement (2, 7). Heart failure is a prevalent syndrome affecting millions globally and is most commonly associated with low cardiac output. In some rare instances, it can occur in the setting of a high-output state. Elevated cardiac output is related to various diseases, such as obesity, arteriovenous shunts, chronic anemia, sepsis, thyrotoxicosis, Paget's disease, Beriberi heart disease, and chronic hypercapnia. The primary pathophysiological mechanism is reduced systemic vascular resistance due to arterio-venous shunting or peripheral vasodilatation, which results in a fall of arterial blood pressure and subsequently in activation of compensatory mechanisms common for heart failure in general. High-output HF shares symptoms(breathlessness at rest or on exertion, exercise intolerance, fatigue, and fluid retention) and signs (tachycardia, tachypnoea, raised jugular venous pressure, pulmonary rales, pleural effusion, and peripheral edema) with typical heart failure. One notable difference is that patients in high-output states may have a warm periphery, rather than a cold one, due to reduced systemic vascular resistance and vasodilatation. Treatment of high-output HF should be directed at the primary cause of the high-output state, such as anemia, arterio-venous shunts, hyperthyroidism, etc (8).

High-output HF is an evident sign for invasive therapy. The most commonly used form of therapy is embolization, which improves clinical outcomes for high-output HF and related symptoms. The form of treatment is patient-dependent, meaning treatment has to be tailored to the individual patient and their pathophysiological substrate. Sometimes, embolization may be the best in any particular case; other times, if AV fistula is anatomically accessible, surgical resection may be better suited, or it may be needed to combine embolization and resection. In the most persistent cases, where new AV fistulas keep maturing and HF reappears and progresses, limb amputation may be the only option to improve quality of life, and sometimes is a life-saving procedure. Determining the best form of therapy for PWS is beyond the scope of this case report and should involve a multidisciplinary team.

Our patient didn't have any signs of HF; as such, cardiac MR was not performed. We believe the main cause of symptoms is CAD, and decreased EF as a result of hibernating myocardium, which are absent on the follow-ups. Purpose of this manuscript is intended to raise awareness of the wider healthcare and scientific community about the possibility of the two conditions appearing together and their deleterious impact on patients' health because these two conditions' effects could combine to propagate one another, forming a kind of “circulus vicious” should one or both be left untreated (1, 2).

## Conclusion

Asymptomatic patients with PWS are not candidates for embolization or other surgical procedures. A meticulous follow-up is required to detect substantial changes that require therapy to avoid serious complications. The best therapy depends on the arterio-venous malformation and the patient's features.

## Data Availability

The original contributions presented in the study are included in the article/Supplementary Material, further inquiries can be directed to the corresponding author.

## Supplementary material

Supplementary Video S1 Shows a huge dilated veins of the arm and pulsatile swelling on the upper arm.
