# Supplementary figures and images for: Case Report: The Parkes-Weber syndrome in the patient who underwent coronary surgery
Source: Front Cardiovasc Med. 2025 Jul 15;12:1479811. doi: 10.3389/fcvm.2025.1479811 (PMC12303869; doi:10.3389/fcvm.2025.1479811)

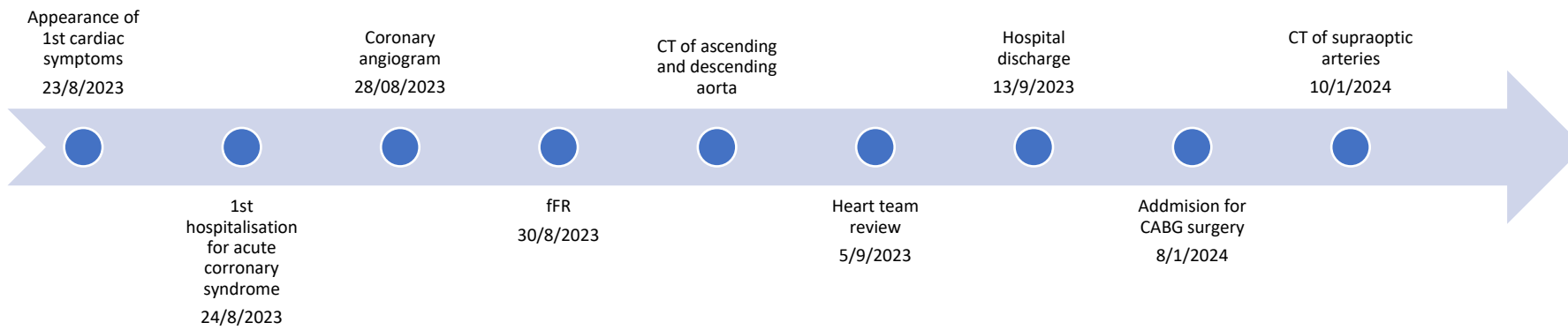

Supplement: Supplementary file 2 [file Image1.pdf]
